# The relation of gelatinase (MMP-2 and -9) expression with distant site metastasis and tumour aggressiveness in colorectal cancer

**DOI:** 10.1054/bjoc.1999.0909

**Published:** 1999-12-08


patient population and appropriate controls. A relatively small number of patients should suffice to indicate whether such mutations are relevant in sporadic BCC.

*T Milan, J Kaprio, PK Verkasalo, CT Jansen, L Teppo and M Koskenvuo, Department of Public Health, University of Turku, Lemminkäisenkatu 1, 20520 Turku, Finland*

**REFERENCES**

Milan T, Kaprio J, Verkasalo PK, Jansén CT, Teppo L, Koskenvuo M (1998) Hereditary factors in basal cell carcinoma of the skin: a population-based cohort study in twins. *Br J Cancer* 1998; **78**: 1516–1520.

Neale MC and Cardon LR (1992) *Methodology for Genetic Studies of Twins and Families*. Kluwer Academic, Dordrecht

### The relation of gelatinase (MMP-2 and -9) expression with distant site metastasis and tumour aggressiveness in colorectal cancer

**Sir,**

We read with great interest the report by Parsons et al (1998) on matrix metalloproteinase (MMP)-2 and -9 expression in gastrointestinal malignancy.

We agree with the authors on the significant role of MMP-2 and -9 in the transformation of a tumour from the benign to the malignant state by enabling the tumour cells to infiltrate blood vessels and lymphatics allowing metastasis to a distant site. However, since the distant site metastasis is an important aspect of the malignancy, it brings a question about the specimens taken from the patients having colorectal cancer, whether they do have metastasis or not. The metastatic colorectal cancers should have been distinguished from the ones that have not metastasized yet. After then it would be much more meaningful to make a comparison between metastatic and non-metastatic groups according to their MMP-2 and -9 expressions in the primary sites.

Additionally, it would be also interesting to find out corresponding results when one considers that some cases are likely to have different organ preferences of metastasis. Moreover, as the MMPs have been implicated in tumour progression (Liotta and Stetler-Stevenson, 1990), with recent evidence suggesting that MMPs are key regulators of the growth of tumours at both primary and metastatic sites (Chambers and Matrisian, 1997), it would be more meaningful to investigate the expressions of MMP-2 and -9 at metastatic sites as well as at primary sites.

On the other hand, all colorectal cancers were classified by the Dukes' staging system as well as other measures. Since only Dukes' stage A, B and C patients were defined in the results, it is highly probable that the original Dukes' staging system (Dukes, 1932) was used in the subdivision of colorectal cancers. Tumour stage 'D' was not included in the original Dukes' staging system and also is not routinely included in Astler–Coller classification (Astler and Coller, 1954) of carcinoma of the colon and rectum which represents modification of classification proposed by Dukes. However, it has become commonly used to represent distant metastasis after addition into Astler–Coller classification by Turnbull et al (1967).

Since stage 'D' is highly related to our topic and was not considered in the study we are not convinced of the conclusion that there is no statistically significant correlation between gelatinase expression and any of the recognized measures of tumour aggressiveness.

Consequently, in the article neither there was a subdivision of colorectal cancers as metastatic and non-metastatic nor tumour stage 'D', which represents distant site metastasis has been included in the staging of colorectal cancers. If the parts of the investigation concerning the relation of gelatinase expression with distant site metastasis and tumour aggressiveness were based upon those above mentioned mainstays much more satisfactory results of the study would be possible to be obtained.

*I. H. Güllü, M. Kurdoğlu, Í. Akalın*
*Institute of Oncology, Hacettepe University, Ankara, Turkey*

**REFERENCES**

Astler VB and Coller FA (1954) The prognostic significance of direct extension of carcinoma of the colon and rectum. *Ann Surg* **139**: 846

Chambers AF and Matrisian LM (1997) Changing views of the role of matrix metalloproteinases in metastasis. *J Natl Cancer Inst* **89**: 1260–1270

Dukes CE (1932) The classification of cancer of the rectum. *J Pathol & Bact* **35**: 323

Liotta LA and Stetler-Stevenson WG (1990) Metalloproteinases and cancer invasion. *Semin Cancer Biol* **1**: 99–106

Parsons SL, Watson S, Collins H, Griffin N, Clarke P and Steele R (1998) Gelatinase (MMP-2 and -9) expression in gastrointestinal malignancy. *Br J Cancer* **78**: 1495–1502

Turnbull RB Jr, Kyle K, Watson FR, et al (1967) Cancer of the colon: the influence of the no-touch isolation technic on survival rates. *Ann Surg* **166**: 420–427

© 2000 Cancer Research Campaign 
